# Hypodermic Medication

**Published:** 1880-08-20

**Authors:** T. J. Tyner

**Affiliations:** Memphis, Tennessee


### HYPODERMIC MEDICATION.

BY T. J. TYNER, M.D., MEMPHIS, TENNESSEE.

On investigating the history of this mode of administering medicines, I find it dates much farther back than has generally been understood.

In the New York Medical Gazette of April, 1870, is an article showing that Drs. Taylor and Washington used it in the New York City Dispensary, as early as 1839. Their method was by puncturing the skin with a lancet, then injecting the fluid with an Anel's syringe.

Dr. Wood, of Edihburgh, used this method in 1843, and supposed he had priority.

Dr. Gross, in his system of Surgery, edition of 1859, while speaking of subcutaneous injections, says :

"The operation which I believe I have been one of the first to perform, is executed with a tight syringe, with a very slender nozzle, which is inserted in a puncture previously made in the skin of the affected part, the subcutaneous cellular tissue being torn up with a common probe to make room for the reception of a drachm of solution of morphia, holding in suspension, from a ½ grain to 1½ grains of the salt, according to the exigencies of the case."

Thus it would seem he was absolutely ignorant of all prior investigations on this subject. His own experience, however, was sufficient to convince him of the advantage of the procedure, for before closing the paragraph he says :

"I believe the subcutaneous injection of morphia will be found highly serviceable in many cases, especially when the disease is distinctly localized, and rebellious to other treatment."

Thus it seems he recognized the fact, that morphia injected under the skin in the cellular tissue, would produce its specific effect when it would not do so in the stomach.

Prof. Trousseau, in his "Lectures on Clinical Medicine," praises very highly the local application of morphine in neuralgia. His mode of application was by removing the cuticle with caustic ammonia, then dressing the raw surface with the salt. In sciatica he punctured the skin, then insinuated under it, small pills of morphia, belladonna, etc., but strange to say, does not mention hypodermic injections.

While the general or systemic effect of hypodermic injections was observed by Dr. Woods and others, yet they attributed its curative influence to the local action of the drug thus given. Mr. Hunter subsequently demonstrated the important fact that by injecting a distant part, the result was the same as when injected at the site of pain; a very valuable discovery, as abscesses are very apt to follow, where the injections are frequently made at the same spot.

However, it was not my intention to discuss the antecedents of the operation, but to endeavor to show that its use has been too much restricted, and that it is not only in extreme and isolated cases it should be used, but in all cases where prompt action is demanded, it is our safest and surest resort. Through the subcutaneous cellular tissue, we do with certainty in a few minutes, what we are uncertain of doing in hours through the stomach.

These are extreme views, but I hope they will be borne out by facts. If it is true, and I believe no one doubts it, who is at all conversant with the subject, that medicines are so potent when subcutaneously given in extreme cases, as for instance, brandy in convulsions due to anæmia of the brain, morphia in intolerant neuralgia, etc., is it not also time that the proper remedies given in the same way in less urgent cases, would be equally if not more effectual?

For the truth of this proposition there are two very cogent reasons: First, the medicine is taken up by the blood; second, without having to undergo the digestive process similar to that which takes place in the stomach, it enters the circulating current unchanged. That it is taken up by the blood has been proven by such experiments as the following:

If a solution of the ext. nux vom. be injected in the subcutaneous areola tissue of the hind leg of a rabbit or dog, it will produce convulsions and death in a few moments, but if another animal is treated in the same way, the blood vessels in the extremity having been previously tied, absorption is much retarded, the poison will find its way into the circulation so slowly, and in such small quantities, that the specific effect will occur only at a late period, or may not manifest itself at all. I refer to Flint, Dalton, and other recent works on Physiology. A very interesting question comes under my notice by force of circumstances; it may have been observed by others, but to me it was not only new, but at the time singular. However, by subsequent experiments, the fact was clearly demonstrated, in individuals whose system had become tolerant to large doses of morphia by the stomach, correspondingly large doses hypodermically, were attended with as much danger as though they had never been addicted to its use. Also, ordinary doses seldom or almost never fail to give relief, when the pain in the same individual had resisted large and repeated doses in the stomach. I have some very interesting notes taken at the time of observation, but it would make this paper too lengthy to read them.

There are conditions of stomach due to nervous impressions, which render digestion impossible for the time. All physiologists have agreed upon this point. The experiments of Dr. Beaumont on St. Martin, (the man with gastric fistula) confirms the above statement.

Irritation of temper would altogether suspend the secretion of gastric juice. Febrile action would produce the same effect. Prof. Dalton says :

"It is very often noticed that when annoyance, hurry or anxiety, occur soon after food is taken, although it may last only for a few moments, the digestive process is not only liable to be suspended for the time, but to be permanently disturbed during the entire day."

This idea may at first sight seem out of place, but a moment's reflection and you will see its bearing upon this subject, and at the same time be convinced of its verity. At all events science has proven it, and I have referred to it, to give more strength to what is to follow, and that is, in reference to the inabilities of the stomach to perform its functions from some cause or disturbance within itself.

While it is true literally, that in excessive pain, excessively large doses of morphine are necessary, yet, in fact I have doubted it for several years, and am now convinced that the pain through reflex action, produces some disturbance in the stomach, which partially suspends its functions, the result of which is, only a part of the morphia is appropriated, the remainder never reaching the blood at all. As proof of this, in severe neuralgia the appetite is as completely suspended as in gastric catarrh. This anorexia does not only last for a few hours, but in some cases for days.

It is a notable fact that we many times fail to get any effect whatever from a drug, which past experience has taught us to rely upon with almost certainty. The reason generally given is worthlessness of the drug, when in reality, it is a failure of the stomach to appropriate it. I have demonstrated this fact with the hypodermic syringe, beyond all reasonable doubt.

In our fall fevers with gastro-hepatic catarrh (which is almost always present), the stomach revolts at everything, and rejects everything almost at the moment it is swallowed, still we write our prescriptions with instructions if the medicine is rejected, repeat it in ten or fifteen minutes, leaving our patient to suffer for hours, whereas, in a few minutes with a decided dose of morphia hypodermically, we make him forget he has a stomach; then with two four-grain doses of quinine in the same way, four or five hours apart, we will as effectually break up his fever as we could with three times that quantity in the stomach, even admitting it be undisturbed and in the perfect performance of its functions.

I hope you will bear with me a few moments longer on this point, as I wish to refer to a case that is very characteristic of the position I have taken; it occurred two years ago.

I was called to see a woman, whom I found to be in labor with her first child (primipara if you please), the head presenting, and tightly wedged in a contracted pelvis. In about three hours she was delivered without instruments, of a living child. I anticipated laceration, but on examination found only slight abrasions of the vulva so trival that I did not regard them as of any consequence. At my next visit, my patient living some distance from my office, I dismissed the case. Two days later I was called to see her; found her temperature 104, pulse 130. At this visit I learned that she had been the subject of fever previous to her confinement. I saw her again the next day at noon; condition unchanged. Complained of soreness, and on examination I was amazed to find that erysipelas, starting from abrasion in the vulva had extended upwards to umbilicus, and downward to knees, presenting a very high degree of inflammation.

In addition to other treatment I prescribed forty grains of quinine with two grains of morphine in twelve pills, two every three hours until six were taken, the remaining six to be given the same way next morning. At my next visit, twenty-four hours later, I found her apparently *in extremis*. Her husband informed me that at eight o'clock that morning she had thrown up all twelve of the pills. He had misunderstood my direction, and given the pills every three hours until all had been taken. I manifested some doubt, and he showed me the vessel in which were the twelve pills coated with a thick tenacious mucus, and but little changed in consistence. Had I not seen them myself, I was prepared to believe him, for she was not in the least under the influence of either the quinine or morphine.

I gave her at once half grain morphine hypodermically, and sent for twelve grains of quinine in solution, and gave at two o'clock one-third in same way, and same quantity at five o'clock. So certain was I that she would die, on taking my leave I told her husband that I would not return, unless he sent for me. I received a message early next morning that my patient was better. Her temperature at 10 a.m. was 100, pulse 86; the erysipelous inflammation was subsiding, and she had drank a cup of tea with relish.

To be brief, her recovery from this time on was uninterrupted. To sum up; the two first pills remained in her stomach eighteen hours. In a word, from the time she took the last two, until they were thrown up, was three hours. Not becoming soluble, of course they could have had no effect; hypodermically, the half grain of morphine gave her relief, and the quinine no doubt saved her life, for I sincerely believe she would have died, had her stomach been the only medium.

I will call your attention to a different phase of the subject, at least somewhat so. Some years ago, there was published in some one of the journals, I don't remember which one now, nor by whom it was written, an article recommending the subcutaneous injection of ergotine in splenic leucæmia. Believing the authority good, I determined to try it upon the first case that presented itself; which I did, and the result was so decisive that I have used it ever since when convenient. My first case was a lady, thirty-six years of age, married, and the mother of two children. She informed me that she had been the victim of an enlarged spleen sixteen years, during which time her health was very bad; at intervals it would somewhat improve, and the enlargement would be slightly reduced, but at no time did it reach its normal size.

When she presented herself for treatment, she appeared more dead than alive, the spleen was enormously enlarged, she had retroversion of the uterus, and a very troublesome dysmenorrhœa. I would not mention these collateral affections, had not the termination of the case led me to believe that the bearing to each other was very intimate.

I at once commenced the use of the ergotine, giving at the same time an iron tonic. I gave the injections over the regions of the spleen on alternate days. Improvement began at once, and in three weeks

I discharged her cured. In all I gave eleven injections, two grains of the drug at each. She bore them well, there being no local disturbance whatever. A few weeks later her husband called and reported her health as perfect. Her complexion had assumed a healthy appearance. She had increased in flesh, and her uterine trouble had disappeared.

Since then, or in all, I have treated thirteen cases, all whites, two of which were under four years of age, eight males and five females. Ten recovered perfectly, two were benefitted, and one could not bear the pain of the operation, although it lasted but fifteen or twenty minutes. He went from under my care, after the second injection.

As an experiment in the meantime, I gave the same drug in three cases, by the stomach persistently, and in increasing doses, without the slightest effect upon the disease. Dr. Abercrombie, of Memphis, very kindly informed me of a case in which he used the ergotine by stomach with negative results. These being one in many of the inscrutable problems in our profession, I would not venture an opinion, unless it has already been given, or explain farther back while speaking of the physiological action of medicine given subcutaneously, thereby entering the blood immediately and in an unchanged state. This is at least plausible if not correct.

As to drugs adapted to hypodermic use, the range is very wide. Either from emergency or experiment, medicines have been brought into daily use, which at first glance would seem not only inadmissible, but dangerous. Bichloride of mercury and chloroform for instance, when applied to the skin produce intense pain, while in the cellular tissue, under the skin, the pain is very slight. The subcutaneous cellular tissue seems to possess a tolerance, or is only to a limited extent susceptible to pain; or perhaps, a more correct solution is, the capillary circulation is so active, the foreign substance is appropriated so quickly it can do no damage.

Were it not for this rapid vital action local phlegmasia would necessarily follow the injection of all irritating substances. In a word, nearly every drug that can be reduced to the soluble state, can be used hypodermically. Of course some care is required in preparing the solution, manipulating the instrument, etc. I have never seen but one case which resulted in abscess. The subject was a druggist with congestive fever; we gave him within twenty-four hours 105 grains of quinine, and an abscess formed at every puncture of the needle. The case occurred at Meridian, Miss., and was treated by Drs. Shackelford, Kline and myself. We were of the opinion that too much acid was used in making the solution. The abscesses were small and got well very quickly; however, some induration remained for several weeks.

In conclusion, I think my experience warrants me in saying that the hypodermic syringe is a most valuable instrument, too much negleted, and that the stomach is in many cases a very unreliable medium to the circulation; and further, that in certain cases we can accomplish a cure with that instrument through the subcutaneous areola tissues, that we could not do through the stomach, although we use precisely the same drug.—*Tenn. Transactions.*

